# Sequestration of Gβγ by deubiquitinated arrestins into the nucleus as a novel desensitization mechanism of G protein–coupled receptors

**DOI:** 10.1186/s12964-022-01013-z

**Published:** 2023-01-19

**Authors:** Xiao Min, Ningning Sun, Shujie Wang, Xiaohan Zhang, Kyeong-Man Kim

**Affiliations:** 1 Department of Pharmacology, College of Pharmacy, Chonnam National University, Gwangju, 61186 Republic of Korea; 2 College of Pharmaceutical Sciences, Guizhou University, Guiyang, 550025 Guizhou China

**Keywords:** Dopamine D2-like receptor, β_2_ adrenoceptor, Desensitization, Src, USP33, Nuclear trafficking

## Abstract

**Background:**

Desensitization of G protein–coupled receptors (GPCRs) refers to a rapid attenuation of responsiveness that occurs with repeated or continuous exposure to agonists. GRK-mediated phosphorylation and subsequent binding with arrestins in the activated receptor cytoplasmic cavity in competition with G proteins has been suggested as the conventional mechanism of desensitization. Along with widely accepted conventional mechanism of desensitization, studies of various GPCRs including dopamine D2-like receptors (D_2_R, D_3_R, D_4_R) have suggested the existence of another desensitization mechanism. In this study, loss-of-function approaches and D2-like receptor mutants that display different desensitization properties were used to elucidate the molecular mechanisms responsible for desensitization.

**Results:**

Desensitization development entailed the signaling cascade composed of Src, PDK1, and Akt, the latter of which in turn interacted with USP33, an arrestin deubiquitinase, to promote arrestin deubiquitination. The deubiquitinated arrestin subsequently formed a complex with Gβγ and translocated to the nucleus via an importin complex, wherein it sequestered Gβγ from the receptor and Gα, thereby attenuating receptor signaling. As in D2-like receptors, both USP33 and importin β1 were involved in the desensitization of the β_2_ adrenoceptor.

**Conclusions:**

In addition to the conventional mechanism of desensitization, which occurs on the plasma membrane and in the cytosol, this study provides a new insight that another desensitization pathway in which nuclear trafficking plays a critical role is operating. It is plausible that multiple, complementary desensitization measures are in place to properly induce desensitization depending on receptor characteristics or the surrounding environment.

**Video Abstract**

**Supplementary Information:**

The online version contains supplementary material available at 10.1186/s12964-022-01013-z.

## Introduction

The regulation of activated G protein–coupled receptors (GPCRs) can occur within different time frames and via several processes, examples of which include uncoupling of receptors and G proteins, sequestration into intracellular compartments, and downregulation [1, 2]. If receptor responsiveness to agonists is rapidly reduced, within, say, a few minutes, it is usually called desensitization. Thus, desensitization can be regarded as a progressive decrease in receptor responsiveness prior to receptor endocytosis or recycling [3, 4].

Desensitization can occur at the receptor level or downstream of the receptor [5]. Desensitization at the receptor level is usually referred to as uncoupling, and is explained by GRK-mediated receptor phosphorylation and subsequent binding of arrestins in steric competition with G proteins to the cavity formed on the cytoplasmic side of activated receptors [6, 7]. Unlike G proteins, whose affinity for the receptor decreases as receptor activation progresses, arrestins maintain their receptor affinity. Hence, in their interactions with activated receptors, arrestins have a competitive advantage over G proteins [8–10]. Thus, arrestin binding to GPCRs sterically precludes further G protein coupling, leading to receptor desensitization [11]. In particular, recent progress in elucidating the structures of GPCR–G protein or –arrestin complexes [12–16] and the electron microscopy–based architecture of GPCR–arrestin complexes [13, 14, 17–20] has revealed an overlapping interface on the receptor for arrestin and the Gα [6].

Dopamine D2-like receptors, namely D_2_R, D_3_R, and D_4_R, act via similar signaling pathways, specifically Gi/o activation, though they have different regulatory properties. For example, D_2_R, but not D_3_R, undergoes GRK2/3-mediated receptor phosphorylation and mediates arrestin translocation to the plasma membrane, in which complementary receptors reside [21]. D_4_R similarly mediates arrestin translocation, albeit less strongly than D_2_R [22]. According to these characteristics, one would expect that D_2_R and D_4_R mediate uncoupling, but that D_3_R does not. However, with respect to desensitization, D_3_R but neither D_2_R nor D_4_R, develops desensitization [23, 24], suggesting that different mechanisms could be involved in the desensitization of GPCRs, depending on receptor or cell environment characteristics.

Given the aforementioned findings from D2-like receptors, we sought in this study to determine the molecular mechanisms involved in GPCR desensitization that could differ from the conventionally accepted mechanism: uncoupling. The pivotal cellular components responsible for desensitization were confirmed by knockdown of those proteins believed to be involved in a regulatory cascade that contributes to receptor desensitization. Collectively, this study revealed that a signaling cascade comprising Src, PDK1, Akt, and ubiquitin-specific protease 33 (USP33) mediate arrestin deubiquitination and acute receptor desensitization by sequestering Gβγ in the nucleus.

## Materials and methods

### Reagents

Dopamine, (−) quinpirole, isoproterenol, forskolin, 4-amino-5-(4-chlorophenyl)-7-(dimethyl ethyl) pyrazolo [3,4-d] pyrimidine (PP2), wortmannin, triciribine, agarose beads coated with monoclonal antibodies against FLAG epitope, rabbit anti-FLAG M2 antibodies (AB_439687), rabbit antibodies against green fluorescent protein (GFP)(AB_439690), and HA antibodies (AB_2610070) were purchased from Sigma-Aldrich Chemical Co. (St. Louis, MO, USA). Goat anti-mouse Alexa Fluor^®^ 555 (AB_2535844), anti-rabbit Alexa Fluor^®^ 555 (AB_2535849), anti-rabbit HRP-conjugated secondary antibodies (AB_10960844), and DAPI were purchased from Thermo Fisher Scientific (Waltham, MA, USA). Antibodies to β-actin (AB_10950489), lamin b1 (AB_10896336), phospho-T308-Akt (AB_331170), phospho-S473-Akt (AB_331163), PDK1 (AB_2236832), phospho-S241-PDK1 (AB_2161134), phospho-Y416-Src (AB_331697), and arrestin2/3 (AB_10547883) were purchased from Cell Signaling Technology (Danvers, Massachusetts, USA). Antibodies to USP33 (AB_2213243), importin β1 (AB_2133993), phospho-ERK1/2 (AB_1125801), and ERK2 (AB_2141292) were obtained from Santa Cruz Biotechnology (Santa Cruz, CA, USA), anti-mouse HRP-conjugated secondary antibodies (AB_10015289) were obtained from Jackson ImmunoResearch (West Grove, PA, USA).

### Cell culture and transfection

Human embryonic kidney 293 (HEK-293, CVCL_0045) cells were obtained from the American Type Culture Collection (Manassas, VA, USA) and cultured in minimal essential medium supplemented with 10% fetal bovine serum (FBS; Thermo Fisher Scientific), 100 units/mL penicillin, and 100 μg/mL streptomycin in a humidified atmosphere containing 5% CO_2_. Simultaneous or individual knockdown of arrestin2 and arrestin3 was conducted by stably transfecting HEK-293 cells with the plasmids containing shRNA against arrestin2 in pcDNA3.0 and/or shRNA against arrestin3 in pcDNA3.1 [24]. The control knockdown (Con-KD) cells for arrestin2 or arrestin3 were established by stable transfection of a plasmid containing scrambled shRNA in pcDNA3.0. Other KD cells, such as Mdm2-KD cells, importin β_1_-KD cells, GRK6-KD, PDK1-KD cells [25, 26], and USP33-KD cells were established by stable transfection of shRNA in pLKO.1 targeted against cellular USP33 (Sigma-Aldrich). Con-KD cells were established by stably expressing scrambled shRNA in pLKO.1.

### Plasmid constructs

Dopamine D_2_R, D_3_R, D_4_R, K149C-D_2_R, C147K-D_3_R, β_2_AR, GRK2-KO-β_2_AR have been described in previous studies [24, 27], as have GFP-Gβ1, Gγ2, arrestin3, NLSX-arrestin3, L395A (NESX)-arrestin3, K11/12R-arrestin3, PDK1, GFP-importin β1, Gβ1*arrestin3, and HA-ubiquitin (HA-Ub). HA-Akt, FLAG-Mdm2, GFP-Mdm2, NLSX-Mdm2 [28], C438A-Mdm2 [29], USP33, T154A-USP33 were prepared via polymerase chain reaction (PCR), and HA-Src have been described previously [30].

### Luciferase report gene assay

Cellular cAMP determinations were based on an indirect method using a reporter plasmid containing the firefly luciferase gene under the control of multiple cAMP-responsive elements (CREs) and pRL-TK as a control vector (Promega Corp., Madison, WI), which has previously been used for determining D_1_R, D_2_R, and D_3_R signaling [31, 32]. This method produces essentially the same results as those obtained based on the use of a direct assay in which [^3^H]-cAMP accumulation is determined via sequential chromatography [31, 33]. Data were normalized by expressing cAMP levels as a percentage of the forskolin-stimulated cAMP for each experiment. Dose–response curves were constructed using Graph Pad Prism (Graph Pad Software; San Diego, CA, USA).

### Induction of desensitization

Desensitization was induced as previously described [24]. Briefly, cells expressing the corresponding D2-like receptors were treated with the agonist for 1–5 min (initial treatment) to trigger desensitization and then washed three times with serum-free medium (5 min per wash) at 37 °C to remove the receptor-bound agonist. The cells were then re-challenged with the agonist to induce a secondary response. The development of desensitization was determined by comparing the amplitudes of the secondary response in vehicle- or agonist-pre-treated cells. Quin( +) and Quin(w/ +) represent the cells treated with quinpirole and washed/re-challenged, respectively.

### Immunoprecipitation and immunoblotting

Cells were initially lysed on a rotation wheel in ice-cold RIPA buffer (50 mM Tris, 150 mM NaCl, pH 8.0, 0.5% deoxycholate, 1% NP-40, 0.1% SDS) for 1 h. at 4 °C. The cell lysates thus obtained were clarified by centrifugation (14,000×*g*) in a microcentrifuge at 4 °C, and the supernatant was mixed with 25 μL of 50% anti-M2 FLAG antibody agarose beads. Having incubated the immunoprecipitated samples overnight at 4 °C with rotation, the anti-M2 FLAG antibody-conjugated agarose beads were washed three times (each for 5 min) with ice-cold washing buffer (50 mM Tris, pH 7.4, 137 mM NaCl, 10% glycerol, 1% NP-40). The immunoprecipitates and cell lysates were analyzed using SDS-PAGE and immunoblotting, with the blots being quantitated using ChemiDoc MP imaging system (BioRad, Hercules, California, USA).

### Detection of arrestin3 ubiquitination

FLAG-arrestin3 and HA-Ub were used to co-transfect HEK-293 cells expressing the corresponding D2-like receptors. The cell lysates were solubilized in RIPA buffer containing 1 mM sodium orthovanadate, 1 mM sodium fluoride, 10 mM N-ethylmaleimide, 5 μg/mL leupeptin, 5 μg/mL aprotinin, and 2 mM phenylmethylsulphonyl fluoride, after which they were immunoprecipitated with FLAG beads. The eluents were analyzed using immunoblotting.

### Immunocytochemistry

Cells were fixed with 4% paraformaldehyde in phosphate-buffered saline (PBS) for 10 min at 20 °C, washed three times with PBS, and then incubated with blocking buffer (1% FBS and 1% BSA in PBS) for 1 h at 20 °C. The cells were then incubated with antibodies against arrestin3 or HA (1:1000 dilution) for 1 h at 20 °C, followed by washing with PBS and incubation with the secondary antibodies (Alexa 555-conjugated antibodies at 1:500 dilution). The immunostained cells were subsequently mounted on slides using Vectashield (Vector Laboratories; Burlingame, CA, USA) and imaged using a laser scanning confocal microscope (TCS SP5/AOBS/Tandem; Leica, Jena, Germany). The images were analyzed using the Fiji version of the image processing software ImageJ and colocalization was analyzed based on Pearson's correlation coefficient (γ value).

### Subcellular fractionation

Subcellular fractions were prepared as reported previously [34]. Briefly, cells were incubated with buffer-1 [10 mM HEPES/KOH (pH 7.8), 1.5 mM MgCl_2_, 10 mM KCl, 0.5 mM dithiothreitol, 0.2 mM phenylmethylsulfonyl fluoride, and 1 mM Na_3_VO_4_] for 20 min. After centrifugation at 2000×*g*, the resulting supernatants were re-centrifuged for a further 10 min at 15,000×*g* and the supernatants were stored as the cytosolic fraction. The pellets remaining after the second centrifugation were washed with buffer-1 for 15 min and centrifuged at 15,000×*g* for 10 min, and the resulting pellets were incubated with buffer-2 [20 mM HEPES/KOH (pH 7.8), 1.5 mM MgCl_2_, 420 mM NaCl, 0.2 mM EDTA, 25% glycerol, 0.5 mM dithiothreitol, 0.2 mM phenylmethylsulfonyl fluoride, and 1 mM Na_3_VO_4_] for 20 min. Following further centrifugation at 24,000×*g* for 10 min, the supernatants were collected as the nuclear fraction. As markers for the cytoplasmic and nuclear fractions, we used actin and lamin b1, respectively.

### Statistical analysis

Studies were designed to create groups of equal size. The statistical analysis was carried out using independent values corresponding to data obtained from different immunoblots or assays. To avoid larger variation between different experiments, the normalized values of immunoblot were averaged and expressed as fold change with the mean value of the control group as 1. Y-axis label in figures represents ‘fold mean of the controls’. The analysis was interpreted using GraphPad Prism 5 software (GraphPad Software Inc., San Diego, CA, USA). All data were expressed as the mean ± SD. Statistical significance was analyzed by using a paired two-tailed Student's *t* test for the two groups or one-way ANOVA with Tukey's post hoc for multiple groups. The post hoc tests were conducted only if the F in ANOVA achieved *P* < 0.05, and there was no significant variance inhomogeneity. Only one threshold for statistical significance (*P* < 0.05) was applied in all analyses.

## Results

### Deubiquitinated arrestin3 and Gβγ translocate to the nucleus under the desensitization conditions of D2-like receptors

It has previously been reported that dopamine D_3_ receptors (D_3_Rs) undergo desensitization in response to cell pretreatment with 100 nM quinpirole (Quin), but do not show the same response when pretreated with 30 nM dopamine (DA). Under desensitization conditions, arrestins are deubiquitinated and form a stable complex with Gβγ to sequester Gβγ from receptors and Gα [35]. To gain a clearer understanding of these processes, we first sought to determine the subcellular regions in which deubiquitinated arrestins and Gβγ form a complex.

**Fig. 1:**
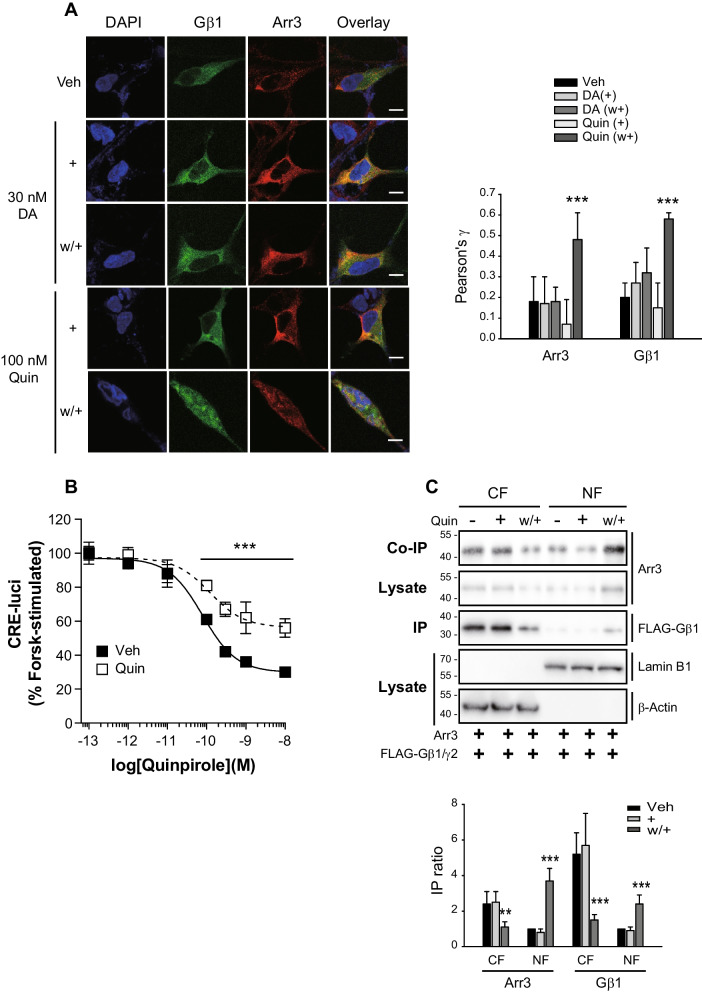
Nuclear translocation of Gβγ and arrestin3 in the desensitization condition of the dopamine D_3_ receptor (D_3_R). Cells were labeled with antibodies to arrestins (1:1000), followed by Alexa 555-conjugated secondary antibodies (1:500). Horizontal bars represent 10 μm. **A** HEK-293 cells were transfected with D_3_R (about 1.8 pmol/mg protein), GFP-Gβ1, Gγ2, and arrestin3. The cells were pretreated with 30 nM dopamine (DA) or 100 nM quinpirole (Quin) for 5 min, washed thrice, and treated again. Colocalization between Gβ1 and DAPI, and between arrestin3 and DAPI, which was quantified by Pearson's correlation coefficient (γ value), significantly increased upon repeated Quin treatment (w + group). Statistical differences among the experimental groups were verified by one-way ANOVA with Tukey's post hoc. ***p < 0.001 compared to other groups (*n* = 9). **B** HEK-293 cells expressing about 1.4 pmol/mg protein D_3_R were used for D_3_R-mediated cAMP production inhibition in the desensitization assay. Statistical differences between the two groups were verified by student's *t* test for the corresponding drug concentrations. ***p < 0.001 compared to the vehicle-treated group at 10^−10^–10.^−8^ M quinpirole (Quin) (*n* = 3). **C** HEK-293 cells were transfected with about 1.5 pmol/mg protein D_3_R, FLAG- Gβ1, Gγ2, and arrestin3. Cells were pretreated with 100 nM Quin for 5 min, washed thrice, and treated again. Cell lysates were fractionated into cytoplasmic and nuclear fractions according to the protocol described in the Materials and methods, and immunoblotted with antibodies against arrestin3, FLAG, lamin B, and β-actin. In the Western blot analysis of cytoplasmic and nuclear fractions, β-Actin and lamin B1 were employed as markers for the cytoplasmic and nuclear fraction (CF and NF), respectively. **p < 0.01, ***p < 0.001 compared to each Veh group (*n* = 3)

As shown in Fig. 1A, both arrestin3 and Gβγ were observed in the cytoplasm of cells expressing D_3_Rs at rest, and translocated to the nucleus under desensitization conditions (Quin, w +) (Fig. 1B). Subcellular fractionation revealed reductions in the cytosolic levels of Gβ1 and arrestin3, and corresponding increases in Gβ1 nuclear levels (Fig. 1C).

To establish whether the nuclear formation of a Gβγ–arrestin3 complex could serve as a desensitization marker for GPCRs, we used receptors with differing desensitization properties to evaluate correlations between receptors’ location in the nucleus and their desensitization. Unlike D_3_R, neither D_2_R nor C147K-D_3_R, a D_3_R mutant, undergoes desensitization [23, 24]. As expected, C147K-D_3_R failed to mediate the nuclear translocation of Gβγ and arrestin3 (Additional file 1: Fig. S1A and S1B). Similarly, D_2_R was unable to mediate the nuclear accumulation of Gβγ–arrestin3 (Additional file 1: Fig. S1C). In contrast, K149C-D_2_R, a mutant of D_2_R that does undergo desensitization, was observed to facilitate the nuclear localization of the two proteins (Additional file 1: Fig. S1D).

Finally, we utilized the dopamine D_4_ receptor (D_4_R), the desensitization properties of which are dependent on the degree of complex stability between Gβγ and arrestin3. Although D_4_R does not undergo desensitization alone, it can undergo desensitization when co-expressed with a Gβ1 and arrestin3 fusion protein (Gβ1*arrestin3) [27, 36]. Accordingly, whereas D_4_R fails to mediate the nuclear translocation of Gβγ and arrestin3 under desensitization conditions (Additional file 1: Fig. S2B), unlike D_3_R (Additional file 1: Fig. S2A), it can mediate this translocation when co-expressed with Gβ1*arrestin3 (Additional file 1: Fig. S2C).

**Fig. 2:**
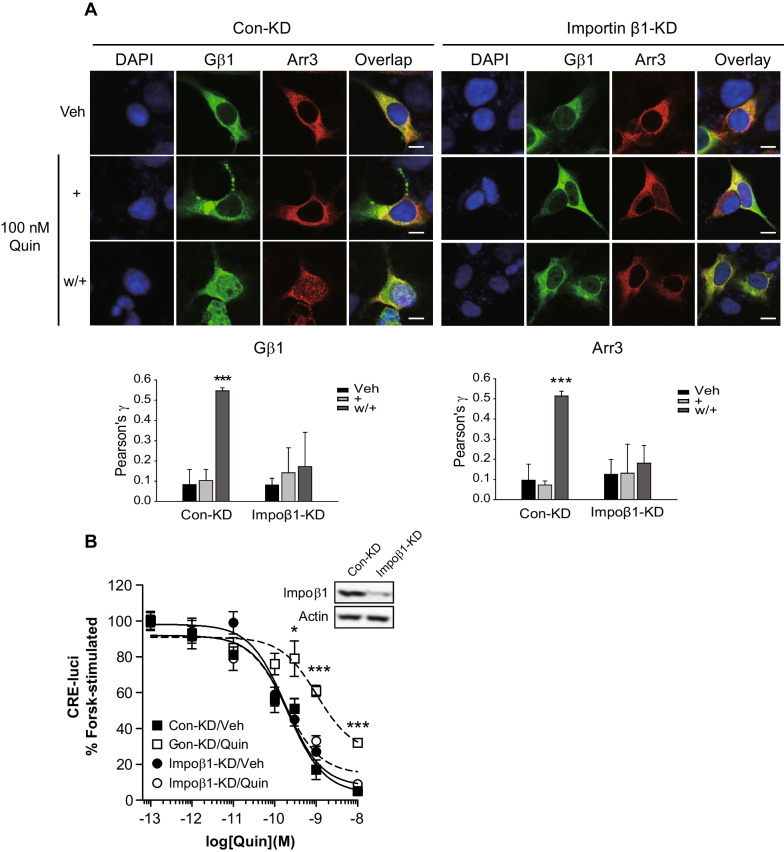
Nuclear entry of Gβγ and arrestin2 through importin is involved in D_3_R desensitization. **A** Control knockdown (Con-KD) and importin β1-KD HEK-293 cells expressing about 1.7 pmol/mg protein D_3_R were transfected with GFP-Gβ1, Gγ2, and arrestin3. Colocalization between DAPI and either arrestin3 or Gβ1 is shown as Pearson's coefficient. ***p < 0.001 compared to the Veh group (*n* = 5). **B** Con-KD and importin β1-KD HEK-293 cells expressing about 1.7 pmol/mg protein D_3_R were used for D_3_R-mediated cAMP production inhibition in the desensitization assay. The cell lysate was immunoblotted with antibodies against importin β1 and actin. Knockdown efficiency for importin β1 was about 85%. Statistical differences among the experimental groups were verified by one-way ANOVA with Tukey's post hoc for the corresponding drug concentrations. *p < 0.05 or ***p < 0.001 compared to the vehicle-treated group at 10^−9.5^–10.^−8^ M quinpirole (Quin) (*n* = 3)

If the nuclear accumulation of Gβγ and arrestin3 is essential for D_3_R desensitization, desensitization should cease upon interruption of nuclear accumulation. To verify this assumption, we utilized importin β1–depleted cells. The importin complex, which comprises importin α and β, mediates the nuclear entry of multiple proteins, including arrestins [37, 26]. We observed that the knockdown of importin β1 inhibited not only the nuclear entry of Gβγ and arrestin3 (Fig. 2A), but also abolished D_3_R desensitization (Fig. 2B).

These results indicate a close association between GPCR desensitization and the nuclear translocation of Gβγ and arrestin3. The nuclear translocation of these two proteins could serve as a desensitization marker for GPCRs, such as D2-like receptors.

### USP33 deubiquinates arrestin3 under desensitization conditions

Given that arrestin deubiquitination is observed under desensitization conditions [35], we next tested whether arrestin3 deubiquitination is actively performed by a deubiquitinase. USP33 (ubiquitin-specific protease 33) is a deubiquitinase for arrestin3 and is involved in the trafficking of some GPCRs [38, 39].

**Fig. 3:**
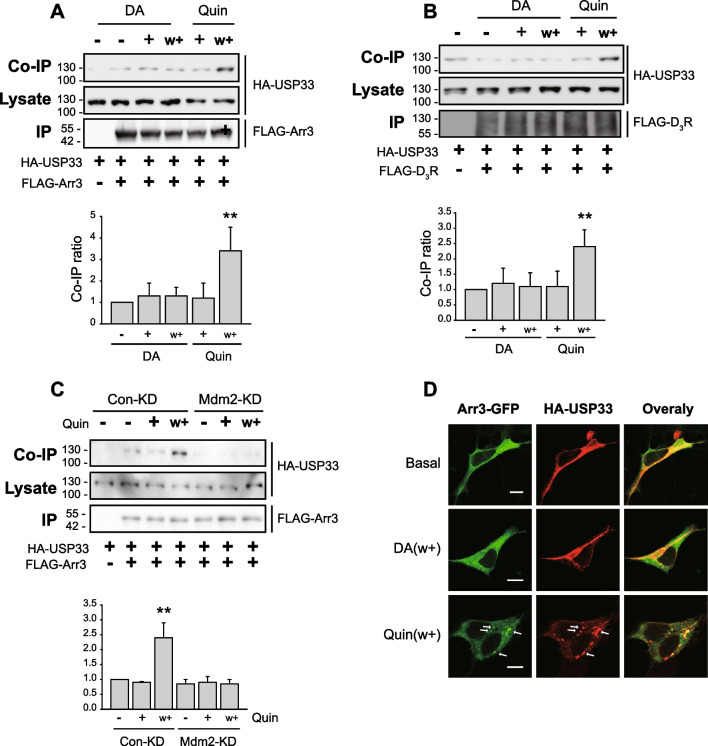
USP33 associates with D_3_R and arrestin3 under desensitization conditions. **A** HEK-293 cells expressing D_3_R (1.9 pmol/mg protein) were transfected with HA-USP33 and FLAG-arrestin3. Cells were treated with 30 nM DA or 100 nM quinpirole (Quin) for 2 min. Co-IP and lysates were immunoblotted with antibodies against HA. IP were immunoblotted with antibodies against FLAG. **p < 0.01 compared to the vehicle-treated group (*n* = 3). **B** HEK-293 cells were transfected with FLAG-D_3_R and HA-USP33. Cells were treated with 30 nM DA or 100 nM Quin for 2 min. Co-IP and lysates were immunoblotted with antibodies against HA. IP were immunoblotted with antibodies against FLAG. **p < 0.01 compared to the vehicle-treated group (*n* = 3). **C** Con-KD and Mdm2-KD cells were transfected with D_3_R, HA-USP33, and FLAG-Arr3. Cells were treated with 100 nM Quin for 2 min according to the desensitization protocol. Co-IP/lysate and IP were immunoblotted with antibodies against HA and FLAG, respectively. **p < 0.01 compared to other groups (*n* = 3). **D** HEK-293 cells stably expressing D_3_R were transfected with arrestin3-GFP and HA-USP33. Data represent results from five independent experiments with similar outcomes. The horizontal bar represents 10 μm

As shown in Fig. 3A and B, interactions between USP33, D_3_R, and arrestin3 remained similar under non-desensitization conditions (30 nM DA, w +), but increased under desensitization conditions (100 nM Quin, w +). In addition, an interaction between USP33 and arrestin3 occurred in an Mdm2-dependent manner (Fig. 3C). Immunocytochemical analysis showed that USP33 formed a complex with arrestin3 under desensitization conditions (Fig. 3D; Quin, w +). These results suggest that USP33 likely locally, rather than globally, deubiquitinates arrestins associated with D_3_R/Mdm2.

**Fig. 4:**
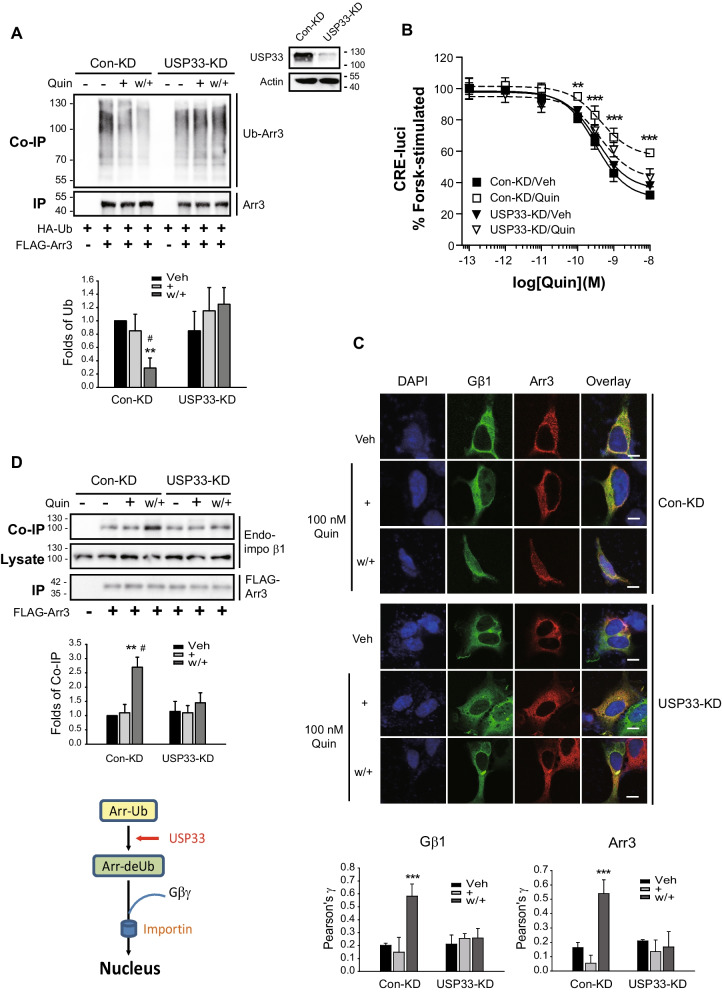
USP33 is involved in the D_3_R desensitization process. **A** Con-KD and USP33-KD HEK-293 cells expressing D_3_R (1.9–2.1 pmol/mg protein) were transfected with HA-Ub and FLAG-arrestin3. Desensitization was induced, and a ubiquitination assay was conducted. The cell lysate was immunoblotted with antibodies against USP33 and actin. Knockdown efficiency of USP33 was about 90%. **p < 0.01 and ^#^*p* < 0.05 compared to the Veh and ( +) group, respectively (*n* = 3). **B** Con-KD and USP33-KD HEK-293 cells expressing D_3_R were treated with 100 nM quinpirole (Quin) to induce desensitization. **p < 0.01 or ***p < 0.001 compared to the vehicle-treated group at 10^−10^–10^−8^ M Quin (*n* = 3). **C** Con-KD and USP33-KD HEK-293 cells expressing D_3_R were transfected with GFP-Gβ1, Gγ2, and arrestin3. Desensitization was induced and the cells were labeled with arrestin antibodies (1:1000), followed by Alexa 555-conjugated secondary antibodies (1:500). Horizontal bars represent 10 μm. ***p < 0.001 compared to other groups (*n* = 5). **D** Con-KD and USP33-KD HEK-293 cells expressing D_3_R were transfected with FLAG-arrestin3. Co-IP/lysates and IP were immunoblotted with antibodies against importin β1 and FLAG, respectively. **p < 0.01 compared with other groups except the “w + /USP33-KD” group (*n* = 3). ^#^*p* < 0.05 compared to w + /USP33-KD cells. The diagram shows that arrestins deubiquitinated by USP33 interact with Gβγ and enter the nucleus through an importin complex

Knockdown of USP33 abolished the deubiquitination of arrestin3 (Fig. 4A), the desensitization of D_3_R (Fig. 4B), and the nuclear entry of Gβγ and arrestin3 (Fig. 4C). We also found that whereas arrestin3 interacted with importin β1 under D_3_R desensitization conditions in which arrestin3 was deubiquitinated, this interaction was abolished in USP33 knockdown cells (Fig. 4D). These findings indicate that repeated agonist treatment initiates a certain signaling pathway that promotes the USP33-mediated deubiquitination of arrestin3, and that this deubiquitinated arrestin3 subsequently undergoes complexation with Gβγ, which in turn translocates to the nucleus via an importin complex (Fig. 4D, diagram).

### Akt facilitates the interaction between USP33 and arrestin3

Akt has been shown to interact with USP14, a deubiquitinase, to enhance the latter’s enzymatic activity [40]. Given that USP33 and USP14 have high sequence homology, and that the ^149^KARGLT^154^ sequence of USP33 possesses a consensus Akt phosphorylation site (RxRxxS/T) [41], it is assumed that Akt would similarly interact with USP33.

**Fig. 5:**
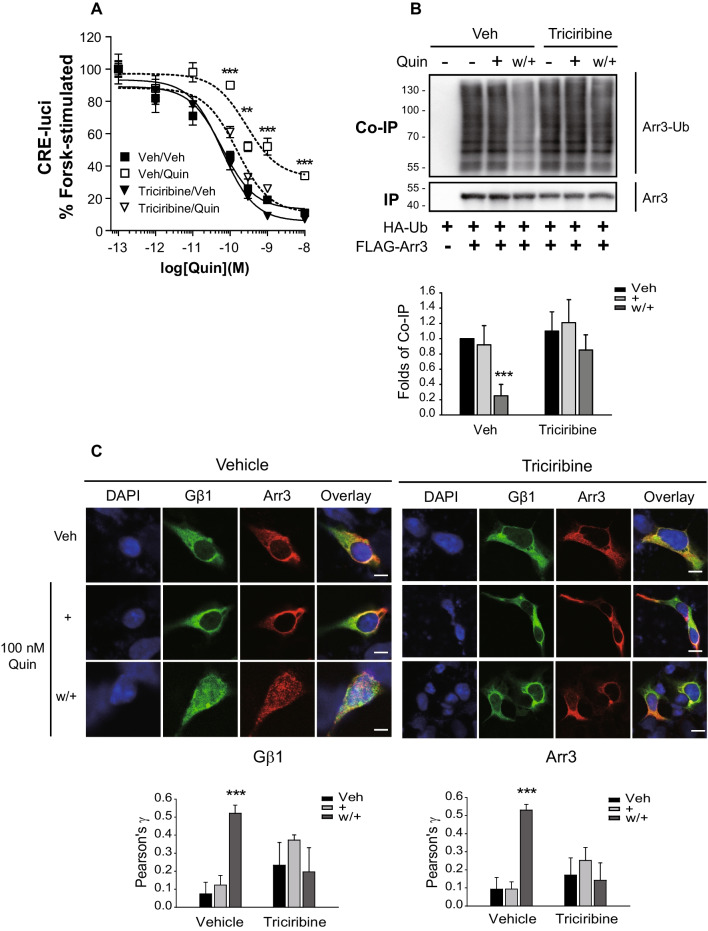
Akt is involved in D_3_R desensitization and arrestin3 deubiquitination. **A** Cell expressing about 1.9 pmol/mg protein D_3_R were pretreated with vehicle or 100 nM triciribine for 30 min, after which desensitization was induced. **p < 0.01 and ***p < 0.001 compared to the vehicle-treated group at 10^−10^–10.^−8^ M quinpirole (Quin) (*n* = 3). **B** HEK-293 cells expressing about 1.8 pmol/mg protein D_3_R were transfected with HA-Ub along with FLAG-arrestin3. The cells were pretreated with either vehicle or 100 nM triciribine for 30 min, after which desensitization was induced. ***p < 0.001 compared to other groups in Veh-treated cells (*n* = 4). **C** HEK-293 cells expressing about 1.5 pmol/mg protein D_3_R, GFP-Gβ1, Gγ2, and arrestin3 were treated with vehicle or 1 μM triciribine for 6 h, after which desensitization was induced. The cells were labeled with antibodies against arrestin (1:1000), followed by Alexa 555-conjugated secondary antibodies (1:500). Horizontal bars represent 10 μm. ***p < 0.001 compared to other groups in vehicle-treated cells (*n* = 5)

**Fig. 6:**
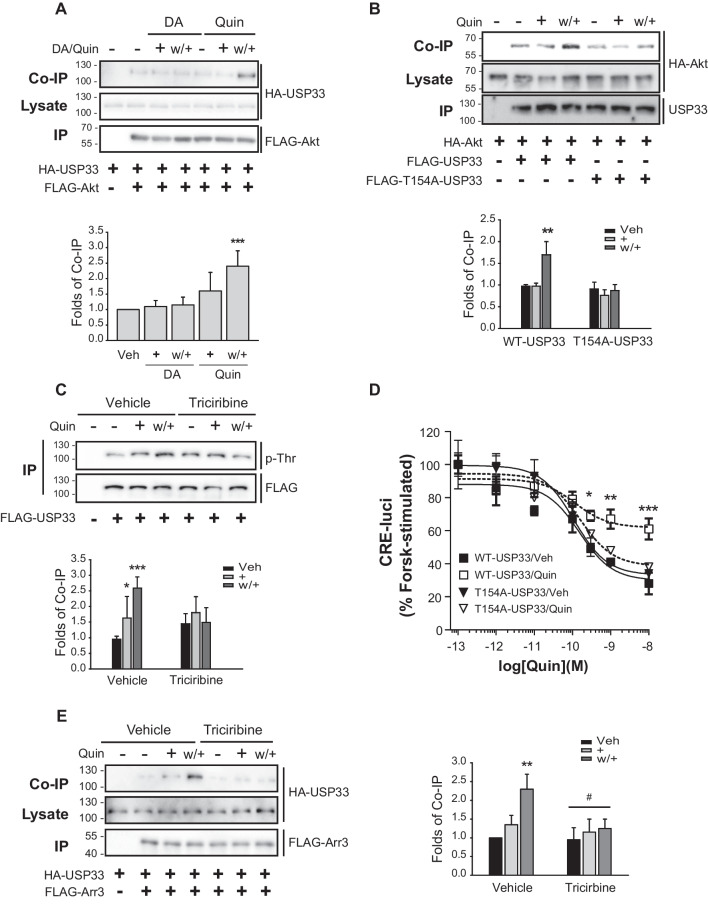
Akt-mediated phosphorylation of USP33 is needed for its interaction with arrestin3. D_3_R expression levels were maintained at 1.8–2.1 pmol/mg protein. **A** HEK-293 cells were transfected with D_3_R, HA-USP33, and FLAG-Akt. The cells were treated with either 30 nM DA or 100 nM quinpirole (Quin) for 2 min. Co-IP/lysate and IP were immunoblotted with antibodies against HA and FLAG, respectively. **p < 0.01 and ***p < 0.001 compared to the Veh group (*n* = 5). **B** HEK-293 cells expressing D_3_R were transfected with HA-Akt, FLAG-USP33, or FLAG-T154A-USP33. Desensitization was induced and the cell lysates were immunoprecipitated with FLAG beads. **p < 0.01 compared to other groups (*n* = 3). **C** HEK-293 cells expressing D_3_R were transfected with FLAG-USP33. The cells were pretreated with 100 nM triciribine for 30 min, after which desensitization was induced. IPs were immunoblotted with antibodies against phospho-threonine and FLAG. *p < 0.05 and ***p < 0.001 compared to Veh/vehicle group (*n* = 3). **D** USP33-KD HEK-293 cells expressing D_3_R were transfected with WT-USP33 or T154A-USP33. *p < 0.05, **p < 0.01, or ***p < 0.001 compared to the vehicle-treated group at 10^−9.5^–10^−8^ M Quin (*n* = 3). **E** HEK-293 cells expressing D_3_R were transfected with FLAG-arrestin3 and HA-USP33. The cells were pretreated with 100 nM triciribine for 30 min, then desensitization was induced. Co-IP/lysates and IP were immunoblotted with antibodies against HA and FLAG, respectively. **p < 0.01 compared to other groups in vehicle-treated cells. ^#^*p* < 0.05 compared to the w + group of vehicle-treated cells (*n* = 3)

As shown in Fig. 5A and B, pretreatment with triciribine, an Akt inhibitor, blocked both D_3_R desensitization and arrestin3 deubiquitination, and also inhibited the nuclear translocation of Gβγ and arrestin3 (Fig. 5C). We observed that whereas the interaction between Akt and USP33 increased under the desensitization conditions (Fig. 6A), this response was absent when we used USP33 harboring a mutated T154 residue, which is a known consensus Akt phosphorylation site (Fig. 6B). Although USP33 was found to be phosphorylated on threonine residue(s) under desensitization conditions, this process was blocked in response to pretreatment with triciribine (Fig. 6C). In addition, D_3_R desensitization, which was abolished following USP33 knockdown, was restored by the co-expression of WT-USP33 but not by the co-expression of T154A-USP33 (Fig. 6D). Consistent with the inhibition of arrestin3 deubiquitination by pretreatment with triciribine (Fig. 5B), this pretreatment was found to block the interaction between arrestin3 and USP33 (Fig. 6E). It can thus be postulated that Akt-mediated phosphorylation of USP33 at T154 plays an essential role in mediating the deubiquitination of arrestin3 and, consequently, the subsequent processes involved in D_3_R desensitization.

### PDK1-mediated Akt phosphorylation at T308 occurs under D_3_R desensitization conditions

**Fig. 7:**
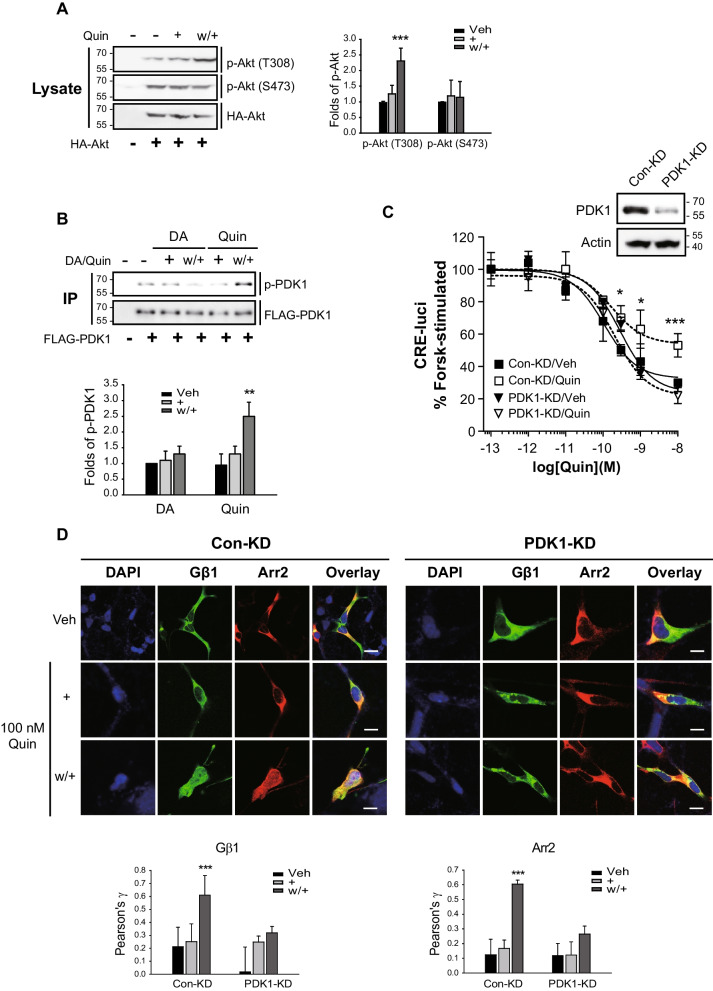
PDK1-mediated phosphorylation of Akt at T308 is involved in D_3_R desensitization. D_3_R expression levels were maintained at 1.8–2.2 pmol/mg protein. **A** HEK-293 cells expressing D_3_R were transfected with HA-Akt. Desensitization was induced and the cell lysates were blotted with antibodies against phospho-Akt (T308 or S473) and HA. ***p < 0.001 compared to other groups (*n* = 3). **B** HEK-293 cells expressing D_3_R were transfected with FLAG-PDK1. Desensitization was induced and the cell lysates were immunoprecipitated with FLAG beads. IPs were blotted with antibodies against p-PDK1 (S241) and FLAG. **p < 0.01 compared to other groups (*n* = 4). **C** Con-KD and PDK1-KD HEK-293 cells expressing D_3_R were treated with 100 nM quinpirole (Quin) according to desensitization protocol. *p < 0.05 and ***p < 0.001 compared to the vehicle-treated group of Con-KD cells at 10^−9.5^–10.^−8^ M Quin (*n* = 3). The cell lysate was immunoblotted with antibodies against PDK1 and actin. Knockdown efficiency of PDK1 was about 85%. **D** Con-KD and PDK1-KD HEK-293 cells expressing D_3_R were transfected with GFP-Gβ1, Gγ2, and arrestin3. Desensitization was induced and the cells were labeled with arrestin antibodies (1:1000), followed by Alexa 555-conjugated secondary antibodies (1:500). Horizontal bars represent 10 μm. ***p < 0.001 compared to other groups (*n* = 5)

As shown in Fig. 7A, Akt was selectively phosphorylated at T308 under the D_3_R desensitization conditions. Because Akt is phosphorylated at the T308 residue by PDK1 [42], we examined the roles of PDK1 with respect to D_3_R desensitization processes. PDK1 is catalytically activated via phosphorylation at S241 located in the activation loop [43], and, as shown in Fig. 7B, this phosphorylation is selectively enhanced upon the induction of desensitization. In addition, we established that both D_3_R desensitization (Fig. 7C) and the nuclear translocation of Gβγ and arrestin3 (Fig. 7D) were abolished in PDK1-KD cells.

### Src is located upstream of the PDK1-Akt axis

To identify factors lying upstream of PDK1, we examined the involvement of Src and PI3K. Previous work has indicated that Src activates PDK1 via the phosphorylation of tyrosine residues [44] and has identified PI3K as a regulatory component lying upstream of PDK1 [45, 46].

**Fig. 8:**
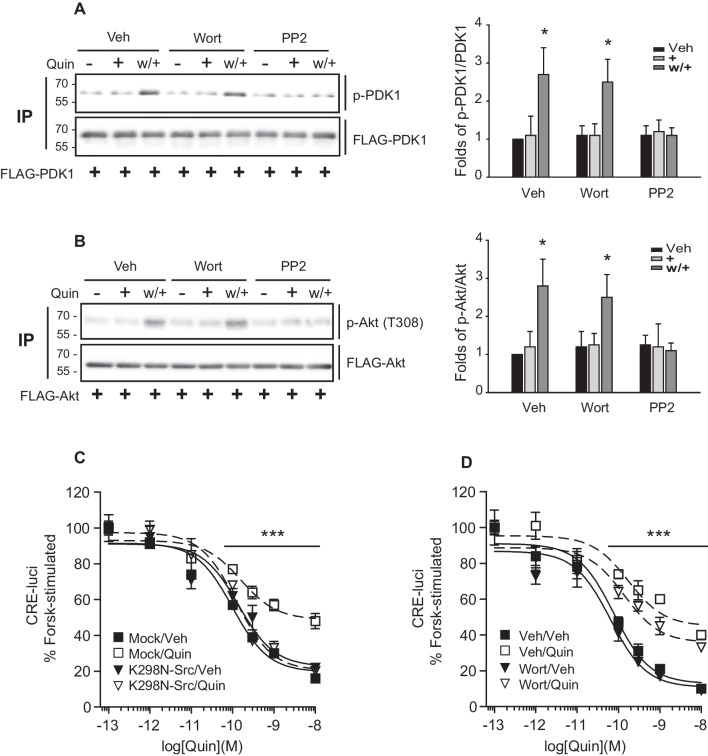
Src mediates PDK1 activation. D_3_R expression levels were maintained at 1.9–2.2 pmol/mg protein. Desensitization was induced as described in the materials and methods. **A** HEK-293 cells expressing D_3_R were transfected with FLAG-PDK1. The cells were pretreated with 1 μM wortmannin or 1 μM PP2 for 30 min. The cell lysates were immunoprecipitated with FLAG beads, and IPs were immunoblotted with antibodies against p-PDK1 (S241) and FLAG. *p < 0.05 compared to corresponding Veh groups (*n* = 3). **B** HEK-293 cells expressing D_3_R were transfected with FLAG-Akt. The cells were treated as in Fig. 8A. IPs were immunoblotted with antibodies against p-Akt (T308) and FLAG. *p < 0.05 compared to corresponding Veh groups (*n* = 3). **C** HEK-293 cells expressing D_3_R were transfected with mock vector or K298N-Src. The cells were treated with 100 nM Quin, according to the desensitization protocol. ***p < 0.001 compared to other groups at 10^−10^–10^−8^ M Quin (*n* = 3). **D** HEK-293 cells expressing D_3_R were pretreated with 1 μM wortmannin for 30 min. The cells were treated with 100 nM Quin, according to the desensitization protocol. ***p < 0.001 compared to corresponding Veh groups at 10^−10^–10.^−8^ M Quin (*n* = 3)

We accordingly demonstrated that pretreatment with the Src inhibitor PP2, but not with the PI3K inhibitor wortmannin, blocked both PDK1 phosphorylation at S241 (Fig. 8A) and Akt phosphorylation at T308 (Fig. 8B). Furthermore, transfection with K298N-Src, a dominant Src negative mutant, suppressed D_3_R desensitization (Fig. 8C), whereas pretreatment with wortmannin had no apparent effect on D_3_R desensitization (Fig. 8D).

**Fig. 9:**
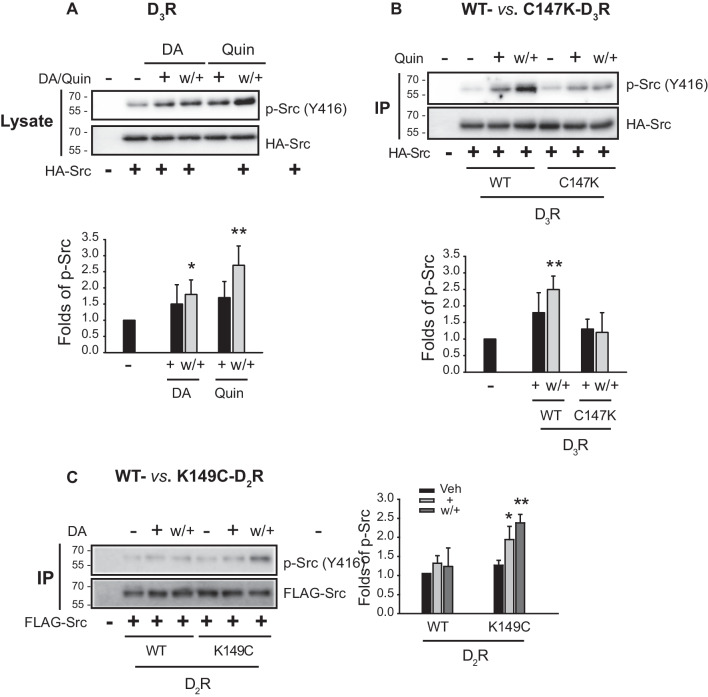
Src activation is accompanied by D_3_R desensitization. Receptor expression levels of D_3_R were maintained at 1.6–1.9 pmol/mg protein. **A** HEK-293 cells expressing D_3_R were transfected with HA-Src. The cells were treated with 30 nM DA or 100 nM quinpirole (Quin). The cell lysates were immunoblotted with antibodies against p-Src (Y416) and HA. *p < 0.05 and **p < 0.01 compared to the Veh group (*n* = 4). **B** HEK-293 cells expressing D_3_R or C147K- D_3_R were transfected with FLAG-Src. The cells were treated with 100 nM Quin. IPs were immunoblotted with antibodies against p-Src (Y416) and FLAG. **p < 0.01 compared to Veh group (*n* = 3). **C** HEK-293 cells expressing D_2_R or K149C-D_2_R were transfected with FLAG-Src. The cells were treated with 10 μM DA. IPs were immunoblotted with antibodies against p-Src (Y416) and FLAG. *p < 0.05 and **p < 0.01 compared to the Veh group (*n* = 3)

The involvement of Src in D2-like receptor desensitization was further confirmed by using D2-like receptor mutants with different desensitization properties (Additional file 1: Fig. S1). Under D_3_R desensitization conditions, while Src activation was observed in the presence of receptors that undergo desensitization, such as D_3_R and K149C-D2R (Fig. 9A and C), no such activation was detected in the presence of D_2_R or C147K-D3R (Fig. 9B and C).

### Arrestin2 and arrestin3 follow the same regulatory pathway

It has been reported that both arrestin2 and arrestin3 are deubiquitinated and form stable complexes with Gβγ in response to D_3_R desensitization [35], indicating that both of these isoforms are likely implicated in D_3_R desensitization. Indeed, we found that simultaneous depletion of cellular arrestin2 and arrestin3 was necessary to suppress D_3_R desensitization (Additional file 1: Fig. S3A and S3B), which may indicate that these arrestin isoforms function collaboratively in promoting the development of D_3_R desensitization. In addition, we established that D_3_R desensitization conditions induce the interaction between arrestin2 and USP33 (Fig. S3C). These findings indicate that the deubiquitination of arrestin2 and arrestin3 is mediated via a single regulatory pathway.

### Desensitization of β_2_ adrenergic receptors can be explained in terms of the USP33-mediated deubiquitination of arrestins and nuclear trafficking of Gβγ/arrestin complex

In order to confirm whether the putative principle of desensitization obtained through this study of D2-like receptors can be applied to other receptors, we examined the β_2_ adrenergic receptor (β_2_AR). The molecular mechanism of GPCR desensitization has been most extensively characterized by employing β_2_AR as a model, and arrestin-mediated physical uncoupling between receptors and G proteins has been established as the fundamental mechanism of GPCR desensitization. We examined whether the novel molecular mechanism observed with D2-like receptors is also involved in β_2_AR desensitization.

**Fig. 10:**
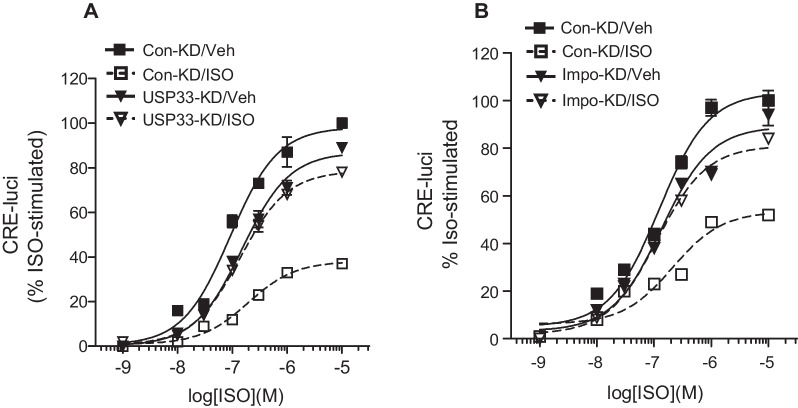
USP33 and importin are involved in the desensitization of β_2_ adrenergic receptors. Cells expressing β_2_AR were used in the desensitization assay performed for cAMP production. Cells were pre-treated with vehicle or 1 μM isoproterenol (ISO) for 5 min, washed three times, and subsequently treated with increasing concentrations of ISO for 5 h. **A** Con-KD and USP33-KD HEK-293 cells expressing 1.3–1.5 pmol/mg protein β_2_AR were used. ***p < 0.001 compared to other groups at 10^–7^–10^–5^ M ISO (*n* = 5). **B** Con-KD and importin β1-KD HEK-293 cells expressing 1.3–1.5 pmol/mg protein β_2_AR were used. ***p < 0.001 compared to other groups at 10^–7^–10^–5^ M ISO (*n* = 5)

As shown in Fig. 10A and B, knockdown of either USP33 or importin β1 abolished β_2_AR desensitization. These results suggest that the principle of desensitization obtained from this study of D2-like receptors—that is, deubiquitination of arrestins via USP33 and nuclear entry of the Gβγ/arrestin complex into the nucleus—also applies to the desensitization of β_2_AR. These findings provide evidence to indicate that the receptor desensitization principle established through our studies of D2-like receptors likely applies to the desensitization of other GPCRs.

## Discussion

In this study, a series of experiments were conducted to elucidate the desensitization mechanism of D2-like receptors, whose desensitization is not clearly explained by the conventional mechanism. The regulatory mechanisms underlying desensitization of D2-like receptors, and to a certain extent β_2_AR receptors, are summarized in Fig. S4.

It has been established that receptors prone to desensitization are constitutively associated with Mdm2 within the cytoplasm in a Gβγ/arrestin-dependent manner, thereby contributing to certain basal levels of arrestin ubiquitination [36]. At this stage, arrestins are likely to be loosely associated with the receptor because the receptors are not yet activated and the intracellular crevices of activated receptors are not open. Thus, in this loose-association state, arrestins are unlikely to compete with G proteins to cause uncoupling.

In response to agonist stimulation, GPCRs are phosphorylated by GRKs, after which GPCRs promote arrestin recruitment to the activated receptors. Many studies have suggested that arrestins compete with G proteins for binding sites in the receptor transmembrane core and sterically preclude further G protein coupling, leading to receptor desensitization [1, 7]. However, follow-up studies considering other receptors, such as the parathyroid hormone receptor or dopamine D2-like receptors, have shown that receptor desensitization might additionally be explained by mechanisms other than the conventional uncoupling mechanism [35, 36, 47]. Specifically, steric hindrance may be induced if arrestins bind to the activated receptor crevice, but may not be induced if arrestins bind to a receptor region other than the crevice.

The signaling cascade involved in the desensitization of GPCRs employed Src to phosphorylate PDK1 at S241, thereby promoting the activation of Akt by facilitating its phosphorylation on T308 (Fig. 5). The phosphorylated Akt subsequently phosphorylated USP33, which was recruited to the D_3_R under desensitization conditions (Figs. 3 and 6). The phosphorylated USP33 deubiquitinated arrestins locally, rather than globally, because USP33 specifically bound to arrestins associated with D_3_R and Mdm2 (Fig. 3). Deubiquitinated arrestins formed a complex with Gβγ that subsequently underwent importin-mediated entry into the nucleus (Figs. 2, Additional file 1: S1, and S2), thereby sequestering Gβγfrom the receptors and Gα located on or near the plasma membrane [35].

Although Gβγ subunits have been found to play key roles in modulating canonical effectors in G proteins at the cell surface, several recent studies have revealed that Gβγ also regulates numerous molecules in distinct subcellular regions [48, 49]. For example, the interactions between Gβγ subunits and partner proteins within the nucleus are modified in response to GPCR activation [50, 51]. Indeed, studies using a primary cerebral cortical neuron culture have revealed that, in response to Gβγ activation, D2-like receptors enhance *IP3R-1* gene transcription by promoting the binding of AP-1 and NFATc4 to the IP3R-1 promoter region [52]. In addition, it has been reported that Gβγ binds to histone deacetylase 5, thereby inhibiting its transcriptional co-repression activity [53]. Considering our findings in the present study, in which we demonstrated that Gβγ translocates to the nucleus in response to repeated agonist stimulation of D2-like receptors, it will be particularly interesting to examine whether the expression of certain genes is regulated concomitantly with receptor desensitization and whether these genes are associated with the progression to pathological states induced in response to heightened receptor activation.

According to studies of D2-like receptors, the modification of arrestin ubiquitination status is a key cellular event underlying receptor desensitization. It is curious why arrestin deubiquitination is needed for Gβγ binding and translocation to the nucleus. Ubiquitination typically enables arrestins to effectively associate with GPCRs, along with other proteins involved in signaling and endocytosis [54]. Accordingly, it can be assumed that the deubiquitination of arrestin alters cellular environments that are optimized for signaling, thereby diminishing signaling efficiency. Studies that elucidate the structural differences between ubiquitinated and deubiquitinated arrestins might provide some fundamental clarifications.

In this study, desensitization with and without steric hindrance seemed to operate separately If arrestin binds to the phosphorylated receptor but does not bind to the receptor’s cytosolic core, the receptor can bind to the G protein even in the presence of arrestin. For these reasons, it is difficult to conclude that steric hindrance is an inapplicable model as a reference in the case in which arrestin is combined with the receptor but receptor desensitization does not occur.

It is particularly noteworthy that the mechanistic principles established in this study, based on our examination of D2-like receptors, apply to the desensitization of β_2_AR. Studies utilizing different approaches almost without exception show that the mechanism of β_2_AR desensitization is the uncoupling between receptors and G proteins [6]. Because desensitization is essential for cell survival, cells may employ multiple, complementary protective mechanisms alongside uncoupling to be utilized depending on receptor characteristics or the surrounding environment.

This study proposed a novel GPCR desensitization mechanism, which differs from the conventional mechanism in which arrestin-induced steric hindrance is a key cellular event. The signaling cascade includes Src, PDK1, Akt, and USP33, and mediates arrestin deubiquitination, leading to receptor desensitization. The principle obtained through this study may enable prediction of the desensitization of certain GPCRs or drugs; above all, it provides new approaches to manipulate desensitization. Considering the functional and pathological implications of GPCR desensitization, the results obtained in this study make crucial contributions to basic research and clinical investigations.

## Supplementary Information

**Additional file 1**. **Fig. S1. **Nuclear translocation of Gβγ and arrestins in the tolerance conditions of D2-like receptors.The cells were labeled with arrestin2/3 antibodies (1:1000), followed by Alexa 555-conjugated secondary antibodies (1:500). Horizontal bars represent 10 μm. HEK-293 cells expressing (**A**) D_3_R, (**B**) C147K-D_3_R, (**C**) D_2_R, or (**D**) K149C-D_2_R were transfected with GFP- Gβ1, Gγ2, and arrestin3. Receptor expression levels were maintained at 1.7–1.9 pmol/mg protein. In D_3_R and C147K-D_3_R groups, the cells were treated with vehicle or 100 nM Quin. In D_2_R and K149C-D_2_R groups, the cells were treated with vehicle or 10 μM DA. ***p<0.001 compared to other groups (n=7). **Fig. S2**. Nuclear translocation of Gβγ and arrestin3 under desensitization condition of dopamine D_4_ receptor. Arrestin2/3-KD cells were transfected with D_3_R, GFP-Gβ1, Gγ2, and arrestin3 (**A**); D_4_R, GFP-Gβ1, Gγ2, and arrestin3 (**B**), or D_4_R, Gγ2, and Gβ1*arrestin3 (**C**). Receptor expression levels were 1.7–1.9 pmol/mg protein, and tolerance was induced by repeated treatment with 100 nM Quin for 5 min. Cells were labeled with arrestin2/3 antibodies (1:1000), followed by Alexa 555-conjugated secondary antibodies (1:500). Horizontal bars represent 10 μm. The cell lysate was immunoblotted with antibodies against arrestins and actin. Knockdown efficiency of arrestin2 and arrestin3 was about 90% and 85%, respectively. ***p<0.001 compared to other groups (n=5). **Fig. S3**. Roles and regulation of arrestin2 in D_3_R tolerance. **A** D_3_R was transfected into Con-KD and arrestin2-KD cells, and lysates from Con-KD and arrestin2-KD cells were immunoblotted with antibodies against arrestin2. The Veh-treated groups were significantly different from the Quin-treated groups at treatment concentrations of 10^-9.5^–10^-8^ M (*p*<0.001, n=5). The cell lysate was immunoblotted with antibodies against arrestins and actin. Knockdown efficiency of arrestin2 and arrestin3 was about 90%. **B** D_3_R was transfected into Con-KD and arrestin3-KD cells, and lysates from Con-KD and arrestin3-KD cells were immunoblotted with antibodies against arrestin3. The results for the 10^-10^–10^-8^ M Veh-treated groups were significantly different from those for the Quin-treated groups (*p*<0.001, n=5). **C** HEK-293 cells expressing D_3_R were transfected with FLAG-arrestin2 and HA-USP33. The cells were treated with 30 nM DA or 100 nM Quin according to tolerance protocol. The cell lysates were immunoblotted with antibodies against FLAG and HA. **p<0.01 compared to other groups (n=5). **Fig. S4. Diagram illustrating the molecular processes involved in D**_**3**_**R tolerance**. In basal states, Mdm2 is recruited to the cytoplasm, constitutively ubiquitinating β-arrestins. Under the tolerance condition, Gα and Gβγ subunit dissociate from the receptor. Simultaneously, the receptor mediates signaling through Src, activating PDK1 then Akt through phosphorylation at S241 and T308, respectively. Active Akt interacts with USP33 to increase its enzyme activity, thus deubiquitinating arrestins. Deubiquitinated arrestins interact with Gβγ and enters the nucleus via importin complex, sequestering Gβγ from the receptor and Gα, which reduces receptor signaling efficiency (desensitization).

## Data Availability

The materials available from the corresponding author on request.

## Abbreviations

Akt, PKB: Protein kinase B
AP-1: Activator protein 1
IP3R: Inositol 1,4,5-trisphosphate receptor
Mdm2: Mouse double minute 2
NFAT_C_4: Nuclear factor of activated T cell 4
PDK1: Phosphoinositide-dependent kinase-1
PH domain: Pleckstrin homology domain
RH domain: Regulator of G protein signaling homology domain
SDS-PAGE: Sodium dodecyl sulfate polyacrylamide gel electrophoresis
USP33: Ubiquitin carboxyl-terminal hydrolase 33
