# Supplementary material for: Sequestration of Gβγ by deubiquitinated arrestins into the nucleus as a novel desensitization mechanism of G protein–coupled receptors
Source: Cell Commun Signal. 2023 Jan 19;21:11. doi: 10.1186/s12964-022-01013-z (PMC9854190; doi:10.1186/s12964-022-01013-z)
Supplement: Supplementary file 2 — Additional file 1. Fig. S1. Nuclear translocation of Gβγ and arrestins in the tolerance conditions of D2-like receptors.The cells were labeled with arrestin2/3 antibodies (1:1000), followed by Alexa 555-conjugated secondary antibodies (1:500). Horizontal bars represent 10 μm. HEK-293 cells expressing (A) D3R, (B) C147K-D3R, (C) D2R, or (D) K149C-D2R were transfected with GFP- Gβ1, Gγ2, and arrestin3. Receptor expression levels were maintained at 1.7–1.9 pmol/mg protein. In D3R and C147K-D3R groups, the cells were treated with vehicle or 100 nM Quin. In D2R and K149C-D2R groups, the cells were treated with vehicle or 10 μM DA. ***p<0.001 compared to other groups (n=7). Fig. S2. Nuclear translocation of Gβγ and arrestin3 under desensitization condition of dopamine D4 receptor. Arrestin2/3-KD cells were transfected with D3R, GFP-Gβ1, Gγ2, and arrestin3 (A); D4R, GFP-Gβ1, Gγ2, and arrestin3 (B), or D4R, Gγ2, and Gβ1*arrestin3 (C). Receptor expression levels were 1.7–1.9 pmol/mg protein, and tolerance was induced by repeated treatment with 100 nM Quin for 5 min. Cells were labeled with arrestin2/3 antibodies (1:1000), followed by Alexa 555-conjugated secondary antibodies (1:500). Horizontal bars represent 10 μm. The cell lysate was immunoblotted with antibodies against arrestins and actin. Knockdown efficiency of arrestin2 and arrestin3 was about 90% and 85%, respectively. ***p<0.001 compared to other groups (n=5). Fig. S3. Roles and regulation of arrestin2 in D3R tolerance. A D3R was transfected into Con-KD and arrestin2-KD cells, and lysates from Con-KD and arrestin2-KD cells were immunoblotted with antibodies against arrestin2. The Veh-treated groups were significantly different from the Quin-treated groups at treatment concentrations of 10-9.5–10-8 M (p<0.001, n=5). The cell lysate was immunoblotted with antibodies against arrestins and actin. Knockdown efficiency of arrestin2 and arrestin3 was about 90%. B D3R was transfected into Con-KD and arrestin3-KD cells, and [file 12964_2022_1013_MOESM2_ESM.docx]

**Supplementary Data to:**

**Xiao Min, Ningning Sun, Shujie Wang, Xiaohan Zhang, Kyeong-Man Kim**

**Sequestration of Gβγ by deubiquitinated arrestins into the nucleus as a novel desensitization mechanism of G protein–coupled receptors**

**
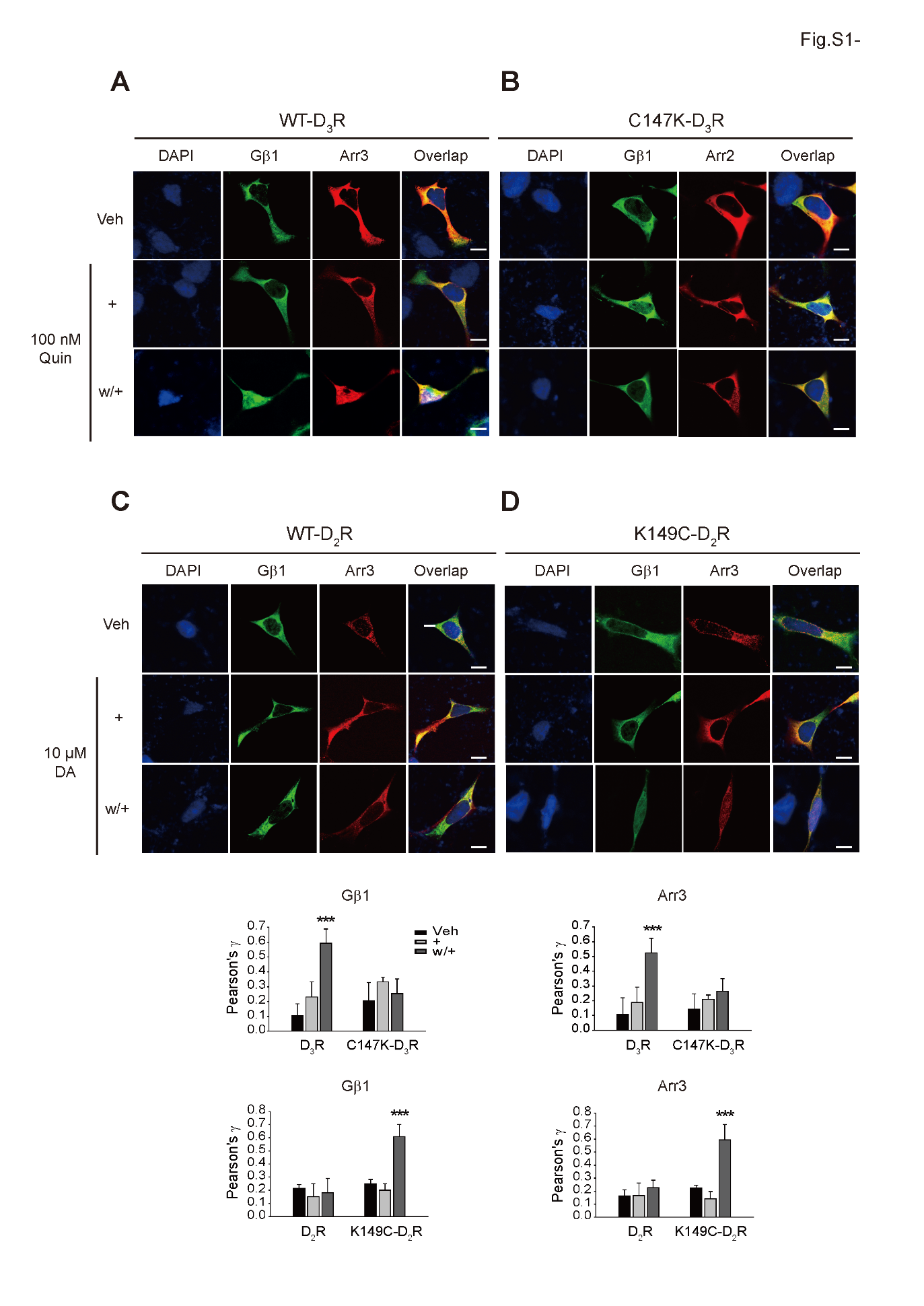
**

**Fig. S1** Nuclear translocation of Gβγ and arrestins in the desensitization conditions of D2-like receptors.

The cells were labeled with arrestin2/3 antibodies (1:1000), followed by Alexa 555-conjugated secondary antibodies (1:500). Horizontal bars represent 10 μm.

HEK-293 cells expressing (**A**) D_3_R, (**B**) C147K-D_3_R, (**C**) D_2_R, or (**D**) K149C-D_2_R were transfected with GFP- Gβ1, Gγ2, and arrestin3. Receptor expression levels were maintained at 1.7–1.9 pmol/mg protein. In D_3_R and C147K-D_3_R groups, the cells were treated with vehicle or 100 nM Quin. In D_2_R and K149C-D_2_R groups, the cells were treated with vehicle or 10 μM DA. ****p*<0.001 compared to other groups (n=7).


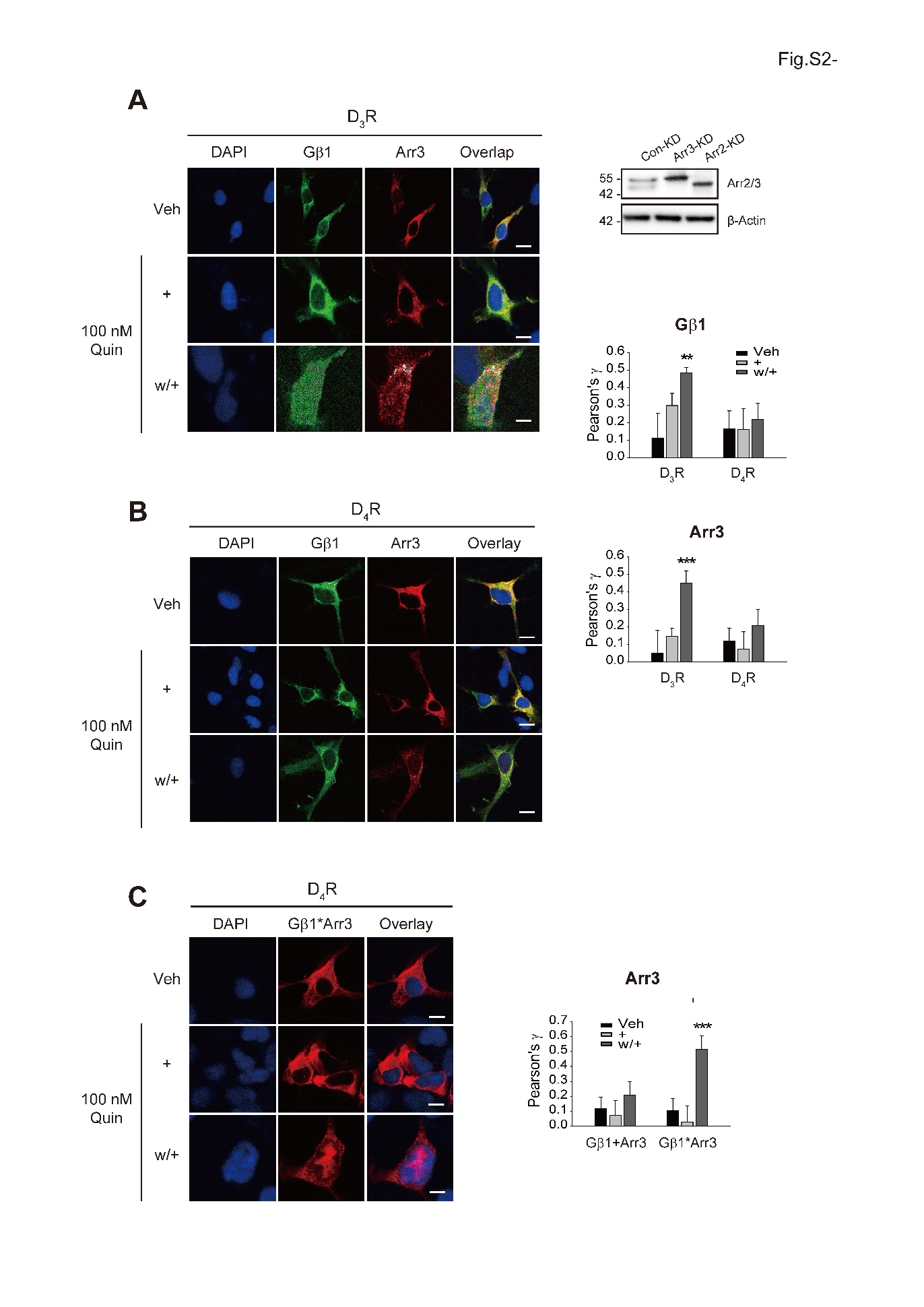


**Fig. S2** Nuclear translocation of Gβγ and arrestin3 under desensitization condition of dopamine D_4_ receptor.

Arrestin2/3-KD cells were transfected with D_3_R, GFP-Gβ1, Gγ2, and arrestin3 (**A**); D_4_R, GFP-Gβ1, Gγ2, and arrestin3 (**B**), or D_4_R, Gγ2, and Gβ1*arrestin3 (**C**). Receptor expression levels were 1.7–1.9 pmol/mg protein, and desensitization was induced by repeated treatment with 100 nM Quin for 5 min. Cells were labeled with arrestin2/3 antibodies (1:1000), followed by Alexa 555-conjugated secondary antibodies (1:500). Horizontal bars represent 10 μm. The cell lysate was immunoblotted with antibodies against arrestins and actin. Knockdown efficiency of arrestin2 and arrestin3 was about 90% and 85%, respectively. ****p*<0.001 compared to other groups (n=5).


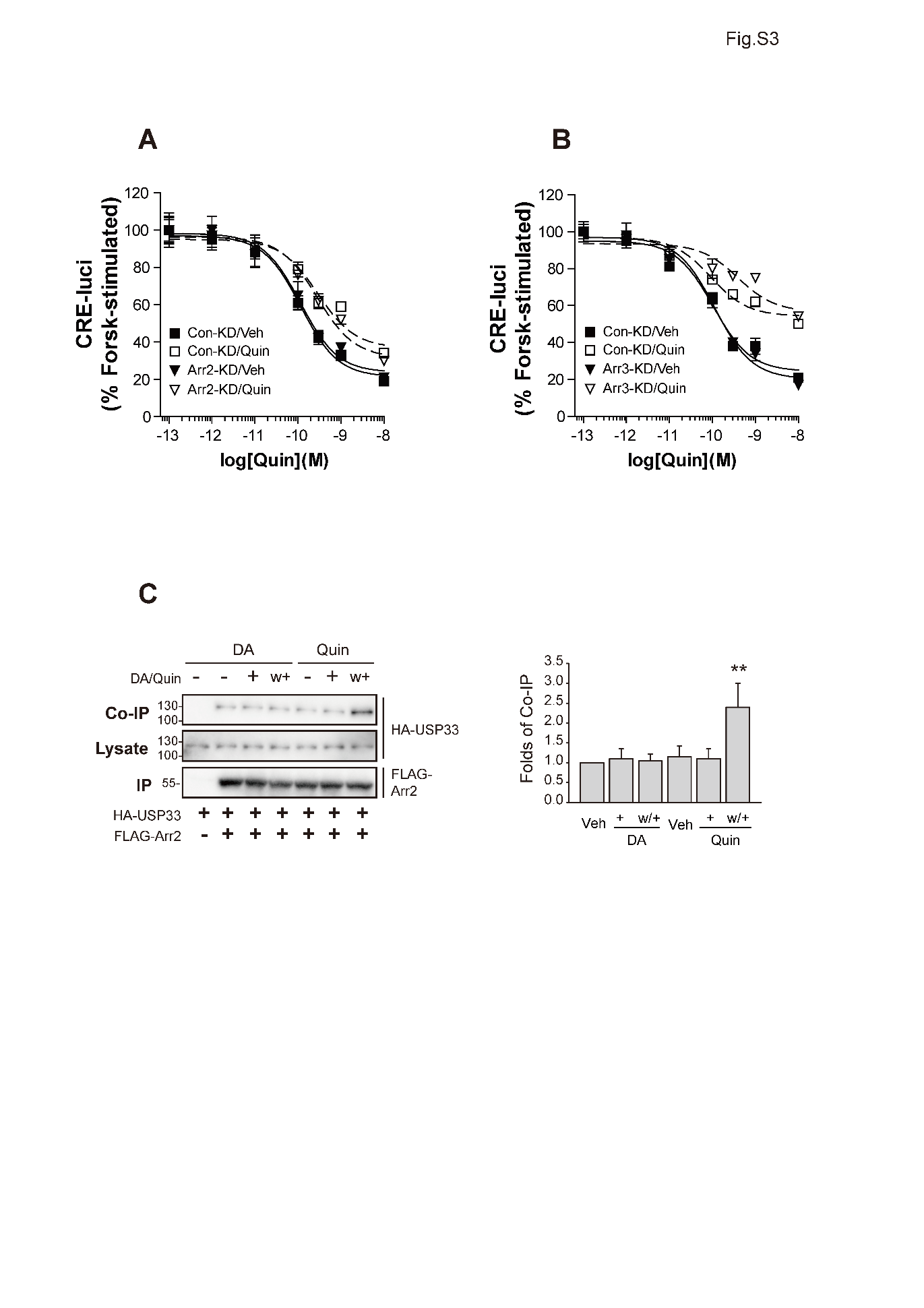


**Fig. S3** Roles and regulation of arrestin2 in D_3_R desensitization.

**A** D_3_R was transfected into Con-KD and arrestin2-KD cells, and lysates from Con-KD and arrestin2-KD cells were immunoblotted with antibodies against arrestin2. The Veh-treated groups were significantly different from the Quin-treated groups at treatment concentrations of 10^-9.5^–10^-8^ M (*p*<0.001, n=5). The cell lysate was immunoblotted with antibodies against arrestins and actin. Knockdown efficiency of arrestin2 and arrestin3 was about 90%.

**B** D_3_R was transfected into Con-KD and arrestin3-KD cells, and lysates from Con-KD and arrestin3-KD cells were immunoblotted with antibodies against arrestin3. The results for the 10^-10^–10^-8^ M Veh-treated groups were significantly different from those for the Quin-treated groups (*p*<0.001, n=5).

**C** HEK-293 cells expressing D_3_R were transfected with FLAG-arrestin2 and HA-USP33. The cells were treated with 30 nM DA or 100 nM Quin according to desensitization protocol. The cell lysates were immunoblotted with antibodies against FLAG and HA. ***p*<0.01 compared to other groups (n=5).


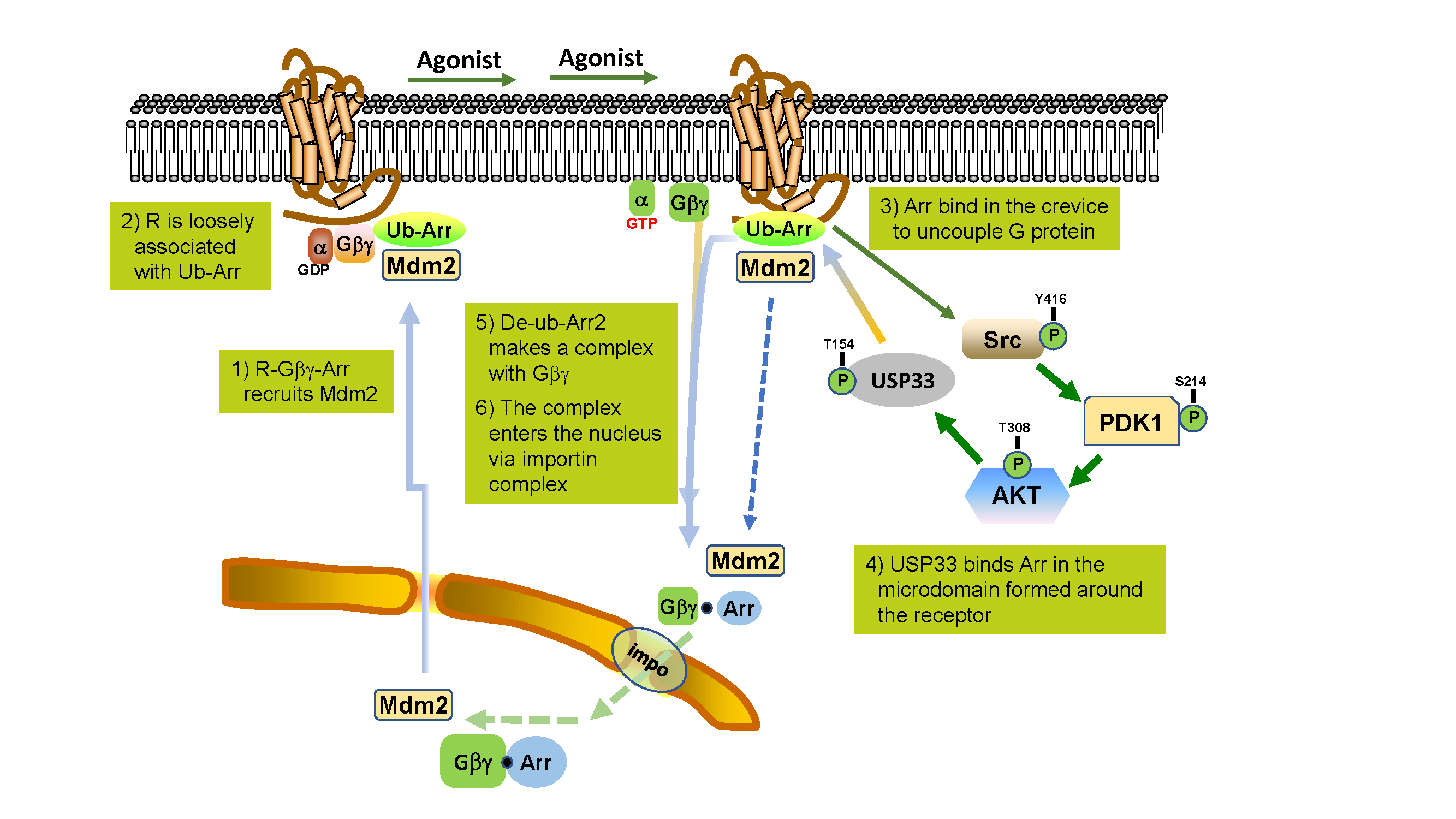


**Fig. S4** Diagram illustrating the molecular processes involved in D_3_R desensitization.

In basal states, Mdm2 is recruited to the cytoplasm, constitutively ubiquitinating β-arrestins. Under the desensitization condition, Gα and Gβγ subunit dissociate from the receptor. Simultaneously, the receptor mediates signaling through Src, activating PDK1 then Akt through phosphorylation at S241 and T308, respectively. Active Akt interacts with USP33 to increase its enzyme activity, thus deubiquitinating arrestins. Deubiquitinated arrestins interact with Gβγ and enters the nucleus via importin complex, sequestering Gβγ from the receptor and Gα, which reduces receptor signaling efficiency (desensitization).
